# Temporal changes in the physical fitness of Chinese adolescents aged 13-18 years: an analysis of eight national successive surveys over three decades

**DOI:** 10.3389/fpubh.2024.1359701

**Published:** 2024-08-19

**Authors:** Hui Ruan, Ruolan Tang

**Affiliations:** ^1^College of Physical Education, Shanxi University, Taiyuan, China; ^2^Institute of Physical Education, Xinjiang Normal University, Urumqi, China

**Keywords:** adolescents, physical fitness, body size, temporal changes, public health

## Abstract

**Objective:**

The aim of this study was to assess temporal changes in physical fitness of Chinese adolescents aged 13–18 years from 1985 to 2019.

**Methods:**

Body size /composition and physical fitness indicators, including body height, weight, body mass index (BMI), speed, power, flexibility, muscular endurance, and cardiorespiratory fitness (CRF), were selected from Chinese boys and girls aged 13–18 years from eight Chinese National Surveillance on Students’ Constitution and Health from 1985 to 2019. Temporal changes in means were estimated by sample-weighted linear regression at the test × sex × age level, and national trends were estimated by a post-stratification population weighting procedure.

**Results:**

Overall mean body height, weight and BMI increased significantly for Chinese adolescents over 34 years. There was a small improvement for boys in speed (Effect size [ES] = −0.21, 95% confidence interval [CI] = −0.44 ~ 0.02), a small improvement for boys in power (ES = 0.24, 95% CI = −0.20 ~ 0.69), a small improvement for girls in flexibility (ES = 0.45, 95% CI = 0.15 ~ 0.76), a moderate decline for boys (ES = -0.53 95% CI = −0.84 ~ −0.21) and a moderate improvement for girls (ES = 0.61, 95% CI = −0.03 ~ 1.26) in muscular endurance, and large declines in cardiorespiratory fitness (CRF) for boys (ES = 0.93, 95% CI = 0.64 ~ 1.21) and girls (ES = 0.93, 95% CI = 0.58 ~ 1.27) from 1985 to 2019. These trends in each component of fitness were more positive for adolescents aged 13–15 years than that of adolescents aged 16–18 years in both sexes, except for girls in flexibility.

**Conclusion:**

The decline in CRF was most pronounced among Chinese children and adolescents from 1985 to 2019, suggesting a future decline in population health that needs attention.

## 1 Introduction

Physical fitness has multiple components, all of which are closely related to the ability to perform physical activity (1). These components span the different functions and structures of body exercise, including musculoskeletal, cardiopulmonary, circulatory, endocrine metabolic and psychoneurological functions (2). Cardiorespiratory fitness (CRF) reflects the ability of the body to transport oxygen from the atmosphere to the mitochondria for physical activity (3). In children and adolescents, CRF was demonstrated to be an independent predictor of all-cause and cardiovascular mortality (4) and was also recognized as an important predictor of executive function (5), academic performance (6), and psychological well-being (7).

Musculoskeletal fitness (MSF) reflects the ability of a muscle or group of muscles to exert force maximally, explosively, or continuously without fatigue (representing maximal muscular strength, power, and muscular endurance, respectively), as well as the ability to move a joint throughout the range of motion (flexibility) (8). In children and adolescents, low MSF is significantly associated with an increased risk of cardiovascular disease and chronic disability due to cardiovascular disease, as well as all-cause mortality in adulthood (9, 10). Speed has been associated with improved motor skills and their performance-related markers of physical fitness (11). Recent studies have pointed out that sport speed mediates the relationship between obesity and cardiometabolic risk factors. In obese children, increased sport speed was associated with lower blood pressure (12).

Adolescence is a critical period for the healthy development of individuals and the accumulation of human resources, and adolescent health and development not only affects itself but also influences adulthood and the next generation (13). Previous studies have shown that physical fitness among Chinese children and adolescents declined from 1985 to 2014, with significant improvements observed only in the first 10 years (2). Several systematic reviews of trends in physical fitness among adolescents have shown that in recent years, muscular strength, measured by grip strength, has improved in some countries (14), and sit-ups, which reflect muscular endurance worsened (15), and standing long jump, which reflects explosive strength, continued to decline (16). Although these studies provide temporal trends in the physical fitness of Chinese adolescents, they lack some components of fitness and the data are relatively old. Moreover, some studies have found that the prevalence of obesity among Chinese adolescents has accelerated in the last decade, with a reversal of the high prevalence of obesity from childhood to adolescence (17). Given an inverted U-shaped relationship between BMI and physical fitness (2, 18), it is necessary to investigate the temporal changes in physical fitness of Chinese adolescents over the past few decades. Due to the currently observed worldwide decline in physical fitness in adolescents and the potential association between physical fitness and nutritional and health status in adulthood, timely monitoring and feedback on physical fitness status is important for improving population health and developing public health policies.

We assumed that some components of physical fitness among Chinese adolescents have improved over the past 34 years, with sex and age disparities. Therefore, this study analyzed the temporal changes in physical fitness among Chinese children and adolescents over the past 34 years by using the Chinese National Surveillance on Students’ Constitution and Health (CNSSCH) surveys in 1985, 1991, 1995, 2000, 2005, 2010, 2014 and 2019.

## 2 Methods

### 2.1 Study design and subjects

Data were derived from eight successive cross-sectional CNSSCHs from 1985 to 2019 (19–26), and we extracted physical fitness test results for Chinese Han adolescents aged 7–18 years (who make up 91% of China’s total population.) The CNSSCH is a national largest and most representative survey of students’ physical fitness and health that is conducted approximately every 5 years using a multistage stratified cluster sampling methodology that maintains consistent sampling and assessment methods throughout each survey year. Twenty-nine provinces/autonomous regions/municipalities (34 in total) were included in the survey in 1985, except for Hong Kong, Macao, Taiwan, Hainan and Chongqing. Hainan has been included in the survey since 1991, Chongqing has been included in the survey since 2000, and Qinghai did not participate in the 1995 survey. Detailed sampling procedures have been described in previous studies (2). In brief, children and adolescents in all provinces except Tibet have been categorized into three levels (high, medium and low) according to their socioeconomic status since 1985 and then stratified according to their place of residence by urban and rural areas, with at least 50 Han Chinese students in each age group participating in the survey. All participants were grouped by sex and age. From 1985 to 2019, 462,960, 462,960, 272,920, 461,975, 462,387, 462,082, 462,022 and 459,386 boys aged 13–18 years and 461,650, 461,650, 272,361, 460,497, 460,796, 460,738, 460,693 and 459,075 girls aged 13–18 years were tested for height, weight, BMI, speed, power, flexibility, muscular strength and CRF, respectively. The ratio of each age group was approximately 1:1, as detailed in Table 1.

**Table 1:** Sample size of each CNSSCH by test and sex.

Sex	Tests	Survey years							
		1985	1991	1995	2000	2005	2010	2014	2019
Boys	Height (cm)	102,404	35,333	52,303	53,977	58,984	53,824	53,449	52,686
	Weight (kg)	102,404	35,333	52,303	53,977	58,984	53,824	53,449	52,686
	Body mass index (kg/m^2^)				53,977	58,984	53,824	53,449	52,686
	50-m dash (s)	102,404	35,333	52,291	53,894	58,891	53,707	53,321	52,134
	Standing long jump (cm)	102,404	35,333	52,290	53,988	58,946	53,789	53,373	52,264
	Stand/sit-and-reach (cm)	102,404	35,333	52,303	53,537	58,924	53,770	53,368	52,443
	Body pull-ups (reps.)	102,404	35,333	52,303	53,960	58,915	53,651	53,349	52,107
	1,000-m running (s)	102,404	35,333	52,269	52,727	58,302	53,224	53,352	51,775
Girls	Height (cm)	102,149	35,017	52,123	54,153	58,848	53,754	53,432	52,174
	Weight (kg)	102,149	35,017	52,123	54,153	58,848	53,754	53,432	52,174
	Body mass index (kg/m^2^)				54,153	58,848	53,754	53,432	52,174
	50-m dash (s)	102,149	35,017	52,096	54,089	58,675	53,639	53,308	51,524
	Standing long jump (cm)	102,149	35,017	52,104	54,171	58,718	53,668	53,332	51,637
	Stand/sit-and-reach (cm)	102,149	35,017	52,123	53,728	58,696	53,649	53,340	52,036
	Sit-ups 60 s (reps.)	102,149	35,017	52,123	54,145	58,731	53,632	53,336	51,560
	800-m running (s)	102,149	35,017	52,056	53,400	58,567	53,601	53,295	50,990

### 2.2 Measures

#### 2.2.1 Body size/component

Body height was measured by a stadiometer to the nearest 0.1 cm. Participants were barefooted, wearing shorts for boys and shorts and short sleeves for girls, and stood with their backs to a column on the base of the stadiometer with their bodies naturally straight and their eyes looking straight ahead. Body weight was measured by an electronic weight scale to the nearest 0.1 kg. Boys wore shorts and girls wore shorts and short sleeves and both stood barefoot in the center of the weight measuring plate, keeping their bodies steady. BMI = [weight(kg)/height(cm)2].

#### 2.2.2 Speed

Speed was measured by a 50-m run to the nearest 0.1 s. Participants in groups of at least 2, using a standing start, recorded the time it took to run 50 m on a flat, straight track (each track 1.22 m wide). Timing was stopped when the participant’s chest reached the vertical plane of the finish line. Manual timekeeping in seconds, retaining one decimal place and recording by adding “1” to the first decimal place if the second decimal place is not “0.” For example, a reading of 10.11 s should be recorded as 10.2 s.

#### 2.2.3 Power

Power was measured by the standing long jump to the nearest 1 cm and performed in a sandpit or on a flat surface with soft soil. The distance from the starting line to the proximal end of the sandpit must not be less than 30 cm. The participant stands behind the jumping line with his/her feet naturally apart, and his/her toes must not step on the line. They take off at the same time with both feet in place and measure the vertical distance between the start line and the post line of the nearest landing spot. Three attempts will be made by each person and the best score will be recorded.

#### 2.2.4 Flexibility

Flexibility was measured by sit-and-reach to the nearest 1 cm. With the electronic sitting forward bending meter, the participant faces toward the instrument, sits on a cushion, and straightens his/her legs forward; the heels are brought together and stomped on the tester’s baffle. During the test, the examinee keeps his/her hands together, palms down and flat, knees straight, upper body bent forward, and pushes the cursor smoothly forward with the tips of the middle fingers of both hands until it cannot be pushed. The test is performed twice and the maximum value is recorded.

#### 2.2.5 Muscular endurance

Muscular endurance was measured by using, pull-ups for boys aged 13–18 years, and 1-min sit-ups for girls aged 13–18 years.

##### 2.2.5.1 Oblique pull-ups

The staff adjusts or chooses a low-height bar so that the bar is at the same height as the chest (nipple) of the participants. The participant faces the bar and overhand grips the bar with his/her hands shoulder-width apart, with his/her legs on the ground in front of him/her, keeping his/her arms at 90° to his/her trunk, and his/her trunk and legs in a straight line. Then, the bent arm was pulled up, when the jaw could touch or exceed the horizontal bar, the arms were extended to recover. For the completion of one, the total completion time was recorded.

##### 2.2.5.2 Pull-ups

At the beginning of the pull-ups, the participant’s hands are separated shoulder-width apart, positively gripping the bar, and the body is in a straight-armed hanging position. No additional movement is allowed during the pull-up process. When the participant’s jaw exceeds the upper edge of the bar, the participant returns to the starting position, thus completing one pull-up and recording the total number completed times.

##### 2.2.5.3 Sit-ups

The participant lies on a cushion with his/her whole body on his/her back, legs slightly apart, knees bent at an angle of approximately 90°, and fingers crossed behind his/her head. When upping, both elbows touched or exceeded both knees as completion of one sitting. Both shoulder blades must touch the cushion when lying on the back. The number of completions in 1 min was recorded.

#### 2.2.6 CRF

CRF was assessed by 1,000-m running for boys aged 13–18 years and 800-m running for girls aged 13–18 years. 1,000-m running or 800-m running: the time when running 1,000 m or 800 m on a flat runway, accurate to 0.01 s.

A group of trained investigators measured five components of physical fitness according to standardized procedures. Specific testing procedures and details are described in previous studies (2). The same measurement program was used at all survey sites and points in time. All the measurement instruments used were consistent in each survey year and were calibrated before use. The measurement was conducted from March to June from 1985 to 1995 and from September to November in subsequent survey years.

### 2.3 Statistical analysis

Using the published summary dataset, all results for body size/component and physical fitness indicators are expressed as the mean and standard deviation (SD). Temporal changes in body shape and physical fitness were estimated for each test × sex × age group by sample-weighted linear regression. Linear models were used because they naturally summarize overall trends (8). We expressed change as absolute change (i.e., the slope B of the regression), percentage change (% per year, i.e., the slope of the regression as a percentage of the sample-weighted mean of all means in the regression), and standardized (Cohen’s) effect size (ES) (i.e., the slope of the regression divided by the combined SD of all SDs in the regression). The 95% confidence interval (CI) for absolute change was calculated as the slope of the regression ±1.96 standard error (27). From the above calculations, we obtained three annual changes, where the overall absolute change is the absolute annual change multiplied by 34, and the overall relative change, the overall effect size, and their 95% CIs were calculated in the same way. Following the procedure described by Tomkinson et al., (15, 16) we calculated population-weighted mean changes for boys and girls aged 7–18 years by combining test × sex × age-specific changes using stratified population weights. The population weights were derived from the United Nations’ 2000 Chinese sex-age-specific population data (28), with 2000 being close to the median year for the entire period of the survey and the test year included in most studies worldwide (16). To account for the magnitude of changes in the mean, ESs of 0.2, 0.5, and 0.8 were used as thresholds for small, medium, and large, respectively, with ESs of <0.2 considered negligible changes (27). Positive ESs indicate an increase in mean over time, showing a positive trend (opposite for speed and CRF); negative ESs indicate a decrease in mean over time, showing a negative trend (opposite for speed and CRF). All statistical analyses were performed by Excel 2016 and SPSS 27.0, and graphs were plotted using GraphPad Prism 9.3.1.

## 3 Results

### 3.1 Changes in body size/composition

**Figure 1:**
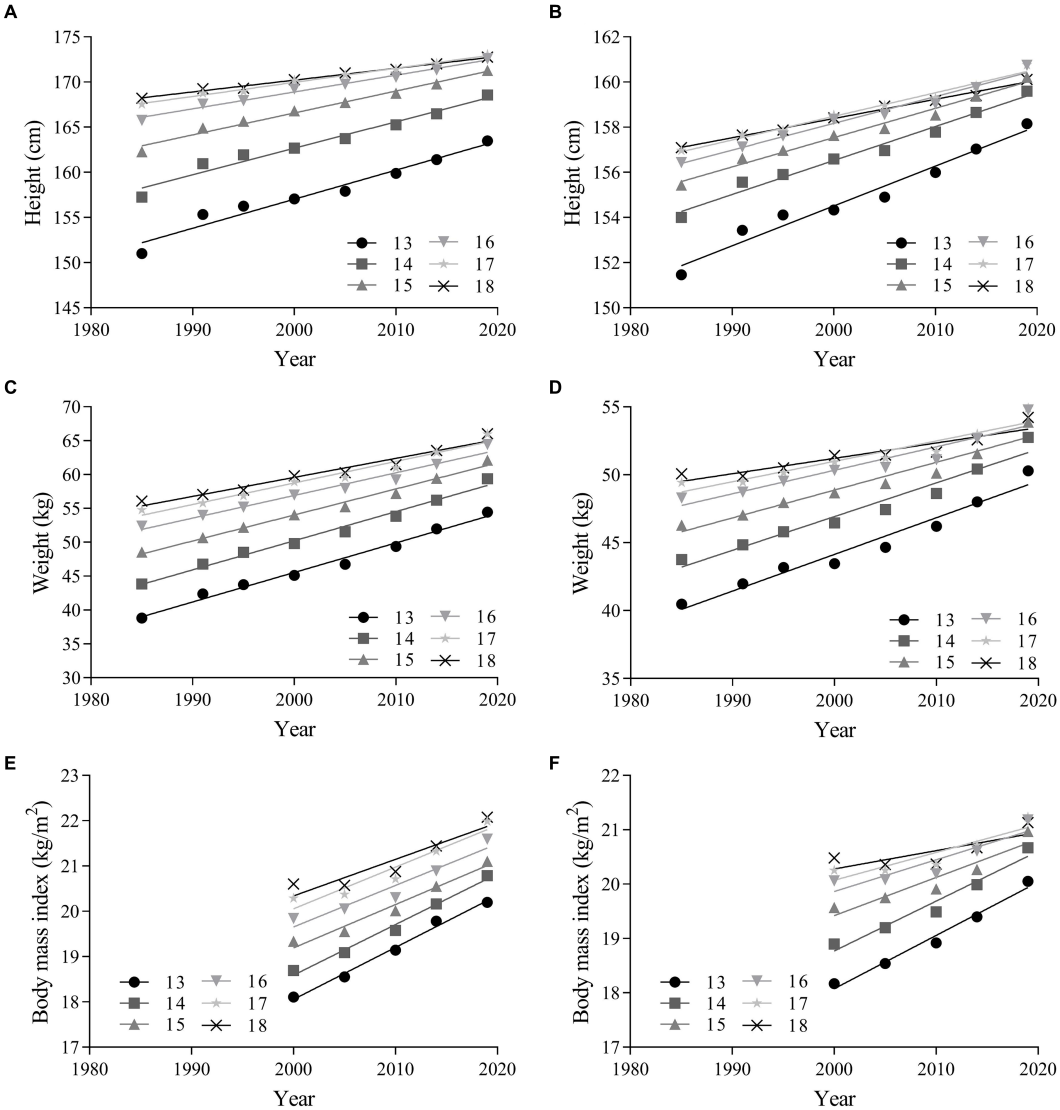
Linear regressions of the mean height, weight and body mass index of Chinese adolescents aged 13–18 years from 1985 to 2019. Boys: **(A,C,E)**; girls: **(B,D,F)**.

The linear regression results in Figure 1 reveals that height, weight and BMI increased in all age groups for both sexes from 1985 to 2019. There was a large increase for overall boys in the mean height (absolute change = 7.97 cm, 95% CI = 7.10 ~ 8.84; percentage change = 4.86, 95% CI = 4.32 ~ 5.39; ES = 1.01, 95% CI = 0.90 ~ 1.11) and weight (absolute change = 12.34 kg, 95% CI = 10.98 ~ 13.70; percentage change = 23.31, 95% CI = 20.80 ~ 25.82; ES = 1.13, 95% CI = 1.00 ~ 1.25) from 1985 to 2019. There was a large increase for overall boys in the mean BMI (absolute change = 3.35 kg/m2, 95% CI = 2.55 ~ 4.15; percentage change = 16.67, 95% CI = 12.75 ~ 20.59; ES = 0.98, 95% CI = 0.74 ~ 1.21) from 2000 to 2019. According to the ESs, the increase was larger in the age group of 13 to 15 years than in the age group of 16 to 18 years. The results for girls were similar to those for boys during the 34 years (Table 2).

**Table 2:** Temporal changes in mean physical size/composition for Chinese adolescents from 1985 to 2019 according to age and sex.

Sexes	Tests	Age categories	*N*	M ± SD	Change in means (95% CI)		
					Absolute	Percent (%)	Standardized ES
Boys	Height (cm)	13 to 15	231,931	162.26 ± 9.59	10.27 (8.96, 11.58)	6.36 (5.55, 7.17)	1.17 (1.02, 1.32)
		16 to 18	231,029	169.82 ± 6.54	5.43 (5.05, 5.82)	3.20 (2.97, 3.43)	0.83 (0.77, 0.89)
		Total	462,960	166.03 ± 9.04	7.97 (7.10, 8.84)	4.86 (4.32, 5.39)	1.01 (0.90, 1.11)
	Weight (kg)	13 to 15	231,926	50.35 ± 12.00	14.22 (13.02, 15.42)	28.61 (26.19, 31.03)	1.24 (1.13, 1.34)
		16 to 18	231,023	58.78 ± 10.23	10.25 (8.72, 11.79)	17.47 (14.86, 20.08)	1.01 (0.86, 1.16)
		Total	462,949	54.56 ± 11.92	12.34 (10.98, 13.70)	23.31 (20.80, 25.82)	1.13 (1.00, 1.25)
	Body mass index (kg/m^2^)	13 to 15	136,722	19.63 ± 3.53	3.67 (3.15, 4.20)	18.75 (16.09, 21.42)	1.05 (0.90, 1.20)
		16 to 18	136,168	20.84 ± 3.33	2.99 (1.89, 4.10)	14.37 (9.06, 19.67)	0.90 (0.57, 1.23)
		Total	272,890	20.24 ± 3.48	3.35 (2.55, 4.15)	16.67 (12.75, 20.59)	0.98 (0.74, 1.21)
Girls	Height (cm)	13 to 15	231,770	156.30 ± 6.32	5.36 (4.77, 5.96)	3.43 (3.05, 3.82)	0.86 (0.77, 0.96)
		16 to 18	229,880	158.45 ± 5.73	3.47 (3.15, 3.79)	2.19 (1.99, 2.39)	0.61 (0.55, 0.66)
		Total	461,650	157.37 ± 6.13	4.46 (3.99, 4.92)	2.84 (2.54, 3.14)	0.74 (0.66, 0.81)
	Weight (kg)	13 to 15	231,785	46.93 ± 8.37	8.04 (6.67, 9.41)	17.29 (14.35, 20.21)	0.98 (0.82, 1.15)
		16 to 18	229,880	51.05 ± 7.26	4.63 (3.34, 5.91)	9.07 (6.56, 11.59)	0.64 (0.46, 0.81)
		Total	461,665	48.98 ± 8.10	6.41 (5.08, 7.74)	13.35 (10.62, 16.09)	0.82 (0.65, 0.99)
	Body mass index (kg/m^2^)	13 to 15	136,481	19.58 ± 3.02	2.97 (2.23, 3.70)	15.22 (11.49, 18.96)	0.99 (0.74, 1.24)
		16 to 18	135,390	20.52 ± 2.73	1.61 (0.61, 2.60)	7.83 (2.98, 12.69)	0.59 (0.22, 0.95)
		Total	271,871	20.05 ± 2.92	2.32 (1.46, 3.17)	11.69 (7.42, 15.96)	0.80 (0.49, 1.10)

Positive trends indicated temporal increases in means and negative trends indicated temporal declines in means. Standardized changes in means of 0.2, 0.5, and 0.8 were used as thresholds for small, moderate, and large, respectively, with an ES of < 0.2 considered to be negligible.

N is the sample size, M is the mean, SD is the standard deviation, CI is the confidence interval and ES is effect size.

### 3.2 Changes in speed

**Figure 2:**
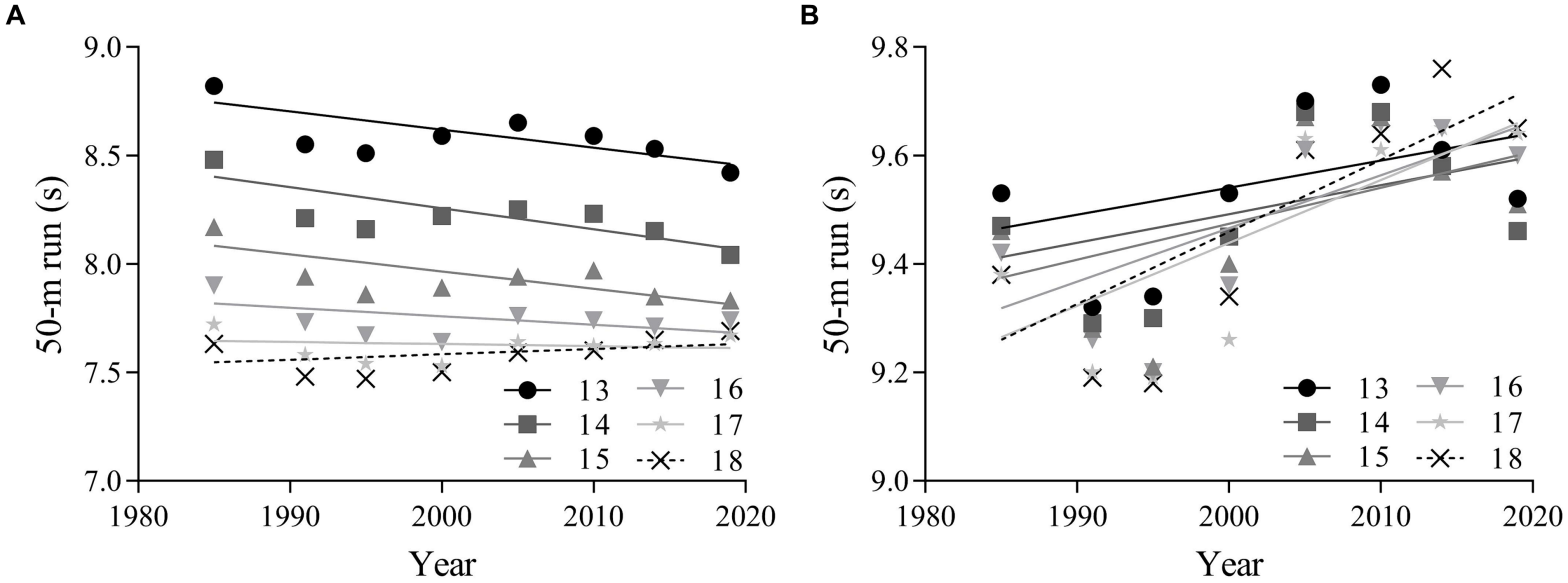
Linear regressions of the 50-m run performance of Chinese adolescents aged 13–18 years from 1985 to 2019. Boys: **(A)**; girls: **(B)**.

The linear regression results in Figure 2 reveals that 50-m run performance of boys and girls in each age group showed different linear trends from 1985 to 2019. There was a small improvement in 50-m run performance for overall boys from 1985 to 2019 (absolute change = −0.17 s, 95% CI = −0.34 ~ 0.01; percentage change = −2.03, 95% CI = −4.20 ~ 0.15; ES = −0.21, 95% CI = −0.44 ~ 0.02). There was a negligible change for boys aged 16–18 years, and a small improvement for boys aged 13–18 years (ES = −0.37, 95% CI = −0.60 ~ −0.13). There was a small decline in 50-m run performance for overall girls (absolute change = 0.29 s, 95% CI = 0.01 ~ 0.56; percentage change = 3.04, 95% CI = 0.13 ~ 5.96; ES = 0.31, 95% CI = 0.01 ~ 0.60), as well as for both age categories (Table 3).

**Table 3:** Temporal changes in mean physical fitness for Chinese children and adolescents from 1985 to 2019 according to age and sex.

Sexes	Tests	Age categories	*N*	M ± SD	Change in means (95% CI)		
					Absolute	Percent (%)	Standardized ES
Boys	50-m dash (s)	13 to 15	231,422	8.27 ± 0.85	−0.29 (−0.48, −0.11)	−3.56 (−5.82, −1.30)	−0.37 (−0.60, −0.13)
		16 to 18	230,553	7.66 ± 0.69	−0.03 (−0.18, 0.13)	−0.33 (−2.40, 1.75)	−0.04 (−0.27, 0.20)
		Total	461,975	7.96 ± 0.83	−0.17 (−0.34, 0.01)	−2.03 (−4.20, 0.15)	−0.21 (−0.44, 0.02)
	Standing long jump (cm)	13 to 15	231,593	198.41 ± 25.84	7.12 (−3.12, 17.36)	3.58 (−1.61, 8.77)	0.30 (−0.13, 0.73)
		16 to 18	230,794	222.98 ± 21.99	4.01 (−6.06, 14.07)	1.81 (−2.71, 6.33)	0.18 (−0.28, 0.64)
		Total	462,387	210.68 ± 26.96	5.64 (−4.52, 15.80)	2.74 (−2.13, 7.61)	0.24 (−0.20, 0.69)
	Stand/sit-and-reach (cm)	13 to 15	231,461	7.90 ± 6.68	−0.63 (−1.89, 0.63)	−8.25 (−24.77, 8.26)	−0.10 (−0.29, 0.10)
		16 to 18	230,621	11.55 ± 7.10	−2.05 (−3.63, −0.48)	−17.64 (−31.29, −4.00)	−0.29 (−0.51, −0.07)
		Total	462,082	9.72 ± 7.13	−1.31 (−2.72, 0.11)	−12.72 (−27.87, 2.43)	−0.19 (−0.39, 0.02)
	Body pull-ups (n)	13 to 15	231,321	3.37 ± 4.07	−1.06 (−2.30, 0.19)	−29.87 (−69.33, 9.60)	−0.26 (−0.57, 0.05)
		16 to 18	230,701	5.93 ± 4.59	−3.75 (−5.22, −2.27)	−62.77 (−87.66, −37.87)	−0.82 (−1.14, −0.50)
		Total	462,022	4.65 ± 4.52	−2.34 (−3.69, −0.98)	−45.51 (−78.04, −12.97)	−0.53 (−0.84, −0.21)
	1,000-m run (s)	13 to 15	229,447	270.45 ± 38.08	31.21 (17.86, 44.56)	11.46 (6.54, 16.38)	0.84 (0.48, 1.20)
		16 to 18	229,939	250.83 ± 32.06	32.83 (26.33, 39.33)	13.10 (10.50, 15.69)	1.03 (0.82, 1.23)
		Total	459,386	260.63 ± 47.30	31.98 (21.88, 42.07)	12.24 (8.43, 16.05)	0.93 (0.64, 1.21)
Girls	50-m dash (s)	13 to 15	231,209	9.51 ± 0.92	0.19 (−0.08, 0.47)	2.01 (−0.87, 4.89)	0.21 (−0.09, 0.51)
		16 to 18	229,288	9.46 ± 0.96	0.39 (0.12, 0.67)	4.17 (1.22, 7.12)	0.41 (0.12, 0.70)
		Total	461,975	9.49 ± 0.94	0.29 (0.01, 0.56)	3.04 (0.13, 5.96)	0.31 (0.01, 0.60)
	Standing long jump (cm)	13 to 15	231,341	161.92 ± 19.45	−3.40 (−11.58, 4.78)	−2.11 (−7.17, 2.94)	−0.18 (−0.60, 0.25)
		16 to 18	229,455	166.75 ± 19.00	0.33 (−8.14, 8.81)	0.20 (−4.88, 5.28)	0.02 (−0.43, 0.46)
		Total	462,387	164.32 ± 19.38	−1.61 (−9.93, 6.71)	−1.01 (−6.07, 4.06)	−0.08 (−0.52, 0.35)
	Stand/sit-and-reach (cm)	13 to 15	231,330	10.28 ± 6.51	3.21 (1.27, 5.15)	31.33 (12.41, 50.25)	0.50 (0.20, 0.79)
		16 to 18	229,408	12.18 ± 6.57	2.68 (0.64, 4.73)	22.06 (5.26, 38.87)	0.41 (0.10, 0.72)
		Total	462,082	11.23 ± 6.61	2.96 (0.97, 4.95)	26.89 (8.99, 44.80)	0.45 (0.15, 0.76)
	1-min sit-ups (n)	13 to 15	231,338	28.54 ± 10.68	5.76 (−0.59, 12.12)	20.14 (−2.14, 42.41)	0.54 (−0.06, 1.13)
		16 to 18	229,355	29.58 ± 10.72	7.48 (0.06, 14.89)	25.27 (0.21, 50.33)	0.70 (0.01, 1.39)
		Total	462,022	29.06 ± 10.71	6.58 (−0.28, 13.44)	22.59 (−1.02, 46.20)	0.61 (−0.03, 1.26)
	800-m run (s)	13 to 15	230,384	250.60 ± 33.58	30.91 (16.80, 45.02)	12.32 (6.69, 17.94)	0.92 (0.50, 1.34)
		16 to 18	228,691	248.68 ± 30.84	28.96 (20.85, 37.07)	11.65 (8.38, 14.91)	0.94 (0.68, 1.20)
		Total	459,386	249.64 ± 32.26	29.98 (18.74, 41.22)	12.00 (7.50, 16.49)	0.93 (0.58, 1.27)

Positive trends indicated temporal increases in means and negative trends indicated temporal declines in means. Standardized changes in means of 0.2, 0.5, and 0.8 were used as thresholds for small, moderate, and large, respectively, with an ES of < 0.2 considered to be negligible.

N is the sample size, M is the mean, SD is the standard deviation, CI is the confidence interval and ES is effect size.

### 3.3 Changes in power

**Figure 3:**
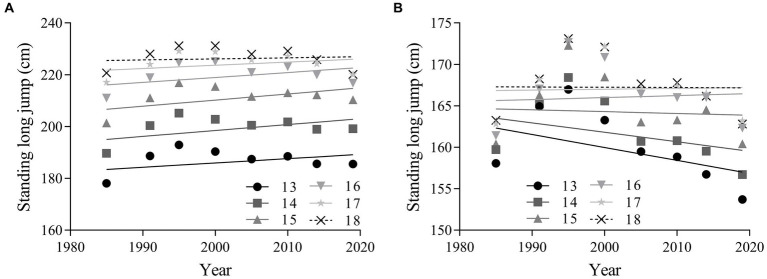
Linear regressions in standing long jump performance of Chinese adolescents aged 13–18 years from 1985 to 2019. Boys: **(A)**; girls: **(B)**.

The linear regression results in Figure 3 reveals that standing long jump performance decreased for girls aged 13–15 and 18 years, increased for girls aged 16–17 years and increased for boys in all age groups from 1985 to 2019. There was a small improvement in standing long jump performance for overall boys from 1985 to 2019 (absolute change = 5.64 cm, 95% CI = −4.52 ~ 15.80; percentage change = 2.74, 95% CI = −2.13 ~ 7.61; ES = 0.24, 95% CI = −0.20 ~  0.69). There was a small improvement for boys aged 13–15 years (ES = 0.30, 95% CI = −0.13 ~ 0.73) and a negligible change for boys aged 16–18 years (ES = 0.18, 95% CI = −0.28 ~ 0.64). There was a negligible change in overall standing long jump performance for girls (absolute change = −1.61 cm, 95% CI = −9.93 ~ 6.71; percentage change = −1.01, 95% CI = −6.07 ~ 4.06; ES = −0.08, 95% CI = −0.52 ~ 0.35), as well as for both age categories (Table 3).

### 3.4 Changes in flexibility

**Figure 4:**
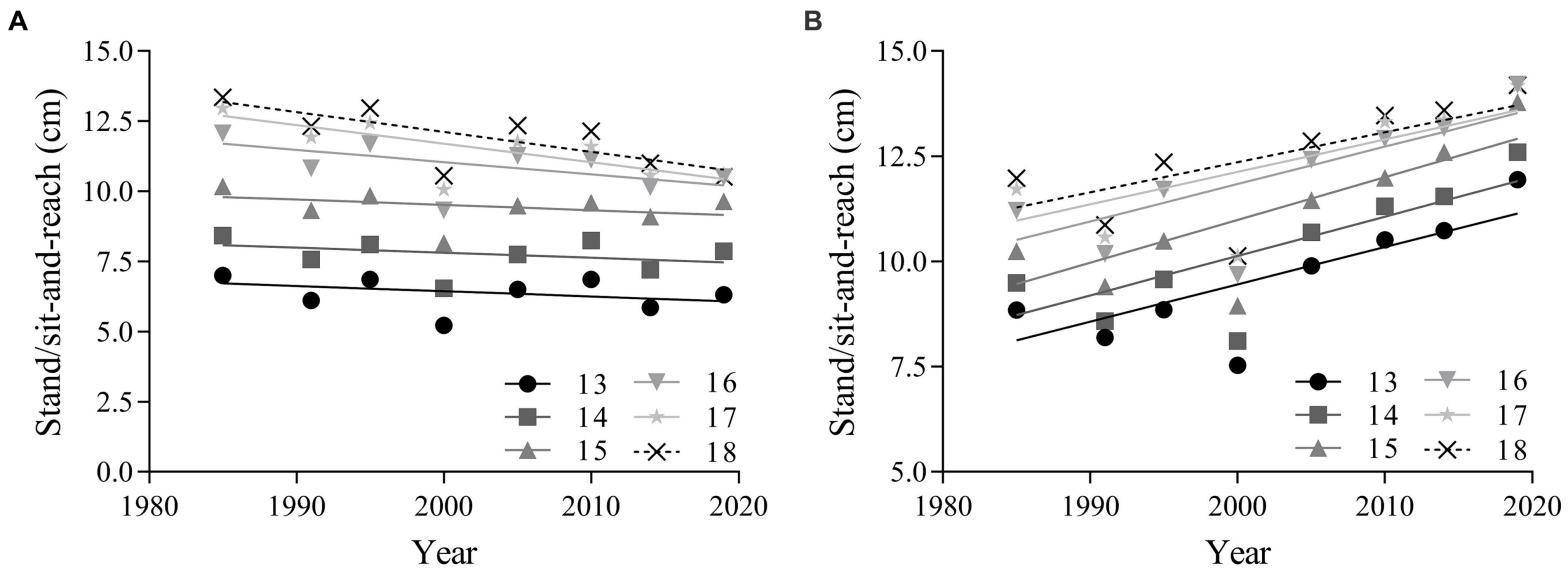
Linear regressions in sit-and-reach performance of Chinese adolescents aged 13–18 years from 1985 to 2019. Boys: **(A)**; girls: **(B)**. From 1985 to 2000, the flexibility test was stand-and-reach. Since 2005, sit-and-reach has been used to measure flexibility.

The linear regression results in Figure 4 reveals that sit-and-reach performance increased for girls in all age groups and decreased for boys in all age groups from 1985 to 2019. There was a negligible change in sit-and-reach performance for overall boys (absolute change = −1.31 cm, 95% CI = −2.72 ~ 0.11; percentage change = −12.72, 95% CI = −27.87 ~ 2.43; ES = −0.19, 95% CI = −0.39 ~ 0.02) and a small decline for boys aged 16–18 years (ES = −0.29, 95% CI = −0.51 ~ −0.07). There was a small improvement in sit-and-reach performance for overall girls (absolute change = 2.96 cm, 95% CI = 0.97 ~ 4.95; percentage change = 26.89, 95% CI = 8.99 ~ 44.80; ES = 0.45, 95% CI = 0.15 ~ 0.76), as well as for both age categories (Table 3).

### 3.5 Changes in muscular endurance

**Figure 5:**
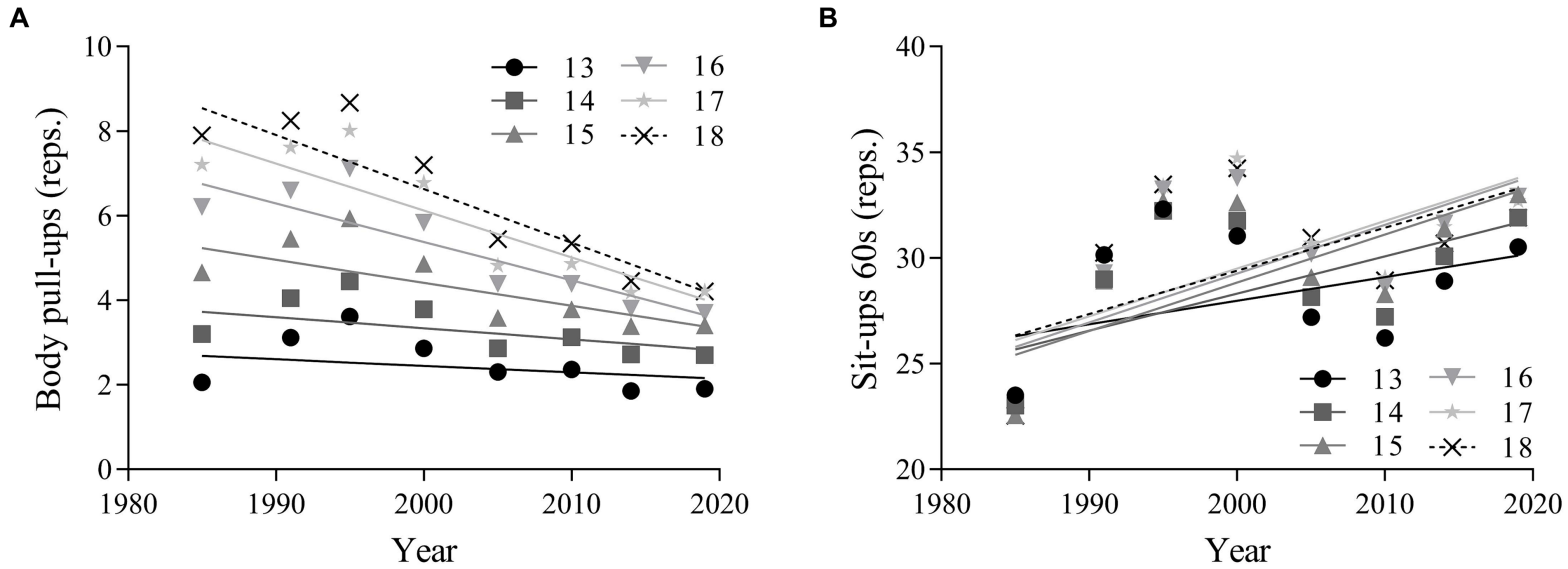
Linear regressions in muscular endurance performance of Chinese adolescents aged 13–18 years from 1985 to 2019. Boys: **(A)**; girls: **(B)**. Muscular endurance is assessed by pull-ups for boys aged 13–18 years, and 1-min sit-ups for girls aged 13–18 years.

The linear regression results in Figure 5 reveals that muscular endurance performance worsened for boys in all age groups and improved for girls in all age groups from 1985 to 2019. There was a moderate decline in pull-up performance for overall boys (absolute change = −2.34 cm, 95% CI = −3.69 ~ −0.98; percentage change = −45.51, 95% CI = −78.04 ~ −12.97; ES = -0.53 95% CI = −0.84 ~ −0.21), a small decline for boys aged 13–15 years (ES = −0.26, 95% CI = −0.57 ~ 0.05), and a large decline for boys aged 16–18 years (ES = −0.82, 95% CI = −1.14 ~ −0.50). There was a moderate improvement in one-minute sit-up performance for overall girls (absolute change = 6.58 reps., 95% CI = −0.28 ~ 13.44; percentage change = 22.59, 95% CI = −1.02 ~ 46.20; ES = 0.61, 95% CI = −0.03 ~ 1.26), as well as for both age categories (Table 3).

### 3.6 Changes in CRF

**Figure 6:**
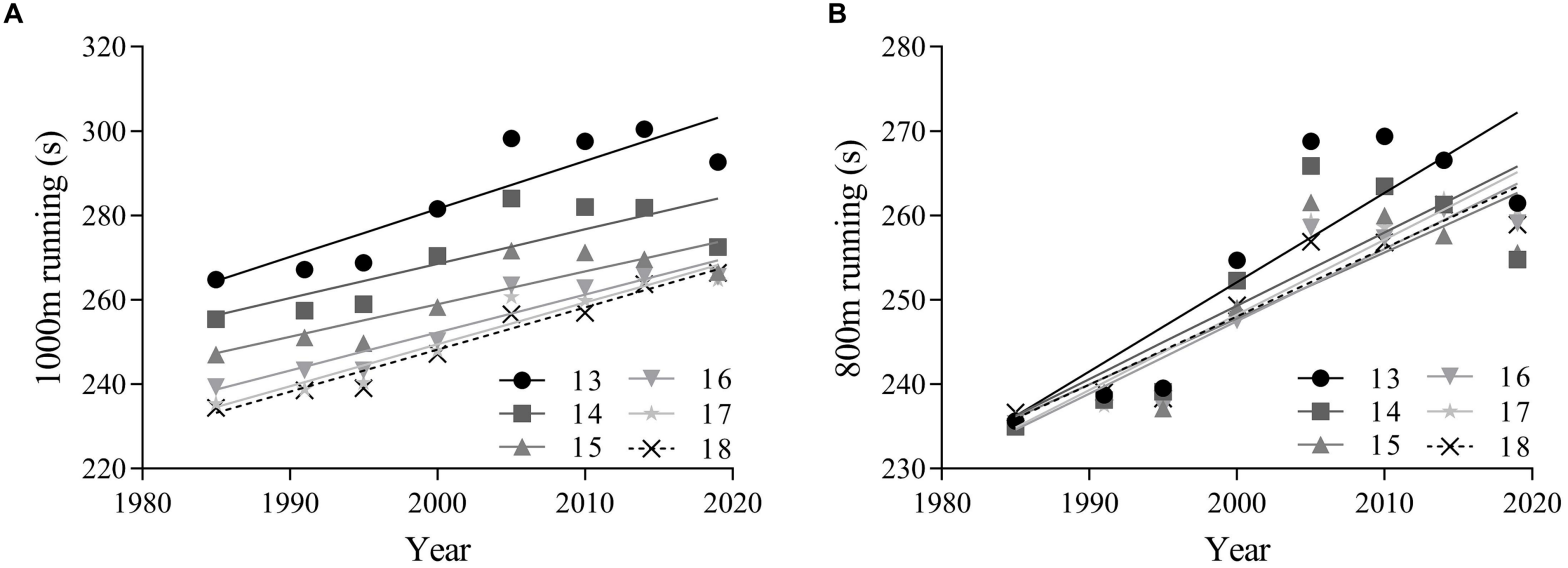
Linear regressions of the endurance running performance of Chinese adolescents aged 13–18 years from 1985 to 2019. Boys: **(A)**; girls: **(B)**. Cardiorespiratory fitness is assessed by 1,000-m running for boys aged 13–18 years, and 800-m running for girls aged 13–18 years.

The linear regression results in Figure 6 revealed that the mean endurance running performance of boys and girls in each age group increased from 1985 to 2019, which represented decline in CRF. There was a large decline in CRF performance for overall boys (absolute change = 31.98 s, 95% CI = 21.88 ~ 42.07; percentage change = 12.24, 95% CI = 8.43 ~ 16.05; ES = 0.93, 95% CI = 0.64 ~ 1.21), as well as for both age categories (13–15 years: ES = 0.84, 95% CI = 0.48 ~ 1.20; 16–18 years: ES = 1.03, 95% CI = 0.82 ~ 1.23). There was a large decline in CRF performance for overall girls (absolute change = 29.98 s, 95% CI = 18.74 ~ 41.22; percentage change = 12.00, 95% CI = 7.50 ~ 16.49; ES = 0.93, 95% CI = 0.58 ~ 1.27), as well as for both age categories (13–15 years, ES = 0.92, 95% CI = 0.50 ~ 1.34; 16–18 years: ES = 0.94, 95% CI = 0.68 ~ 1.20) (Table 3).

## 4 Discussion

This study assessed the temporal changes in physical fitness among Chinese adolescents aged 7–18 years from 1985 to 2019. In addition, changes in body size/composition were also investigated, which may help to understand potential influencing factors (29, 30). The main findings during the 34 years were that (a) mean height, weight and BMI increased significantly for both sexes, (b) there was a small improvement for boys and a negligible change for girls in speed, (c) there was a small improvement for boys and a negligible change for girls in power, (d) there was a negligible change for boys and a small improvement for girls in flexibility, (e) there was a moderate decline for boys and a moderate improvement for girls in muscular endurance, (f) there were large declines in CRF in both sexes, and (g) the trends in each component of fitness were more positive for adolescents aged 13–15 years than that for adolescents aged 16–18 years in both sexes, except for girls in flexibility.

The study found a large decline in CRF for Chinese adolescents over 34 years, which was consistent with other studies in China (2, 31–33). A systematic review by Lang et al. (34) found that CRF levels (measured by 20-m shuttle run) of Chinese children and adolescents aged 9–17 years, when compared with international standards, were at a level of moderate and ranked 25th among 50 countries. A systematic review of trends in physical fitness in children and adolescents in many countries, pooled by Tomkinson et al. showed that global CRF (mainly measured by 20-m shuttle run) worsened from 1975 until it stabilized at the beginning of the 21st century (35–37). Fühner (38) et al. found that CRF (as measured by different tests) improved in children and adolescents globally from 1972 to 1986, then declined continuously until approximately 2010, stabilized and even improved again. In summary, there is evidence that global CRF in children and adolescents declined and stabilized by the turn of the century, especially in Western industrialized countries. However, several studies that included the most recent 10-year span found that CRF in children and adolescents continued to decline (39, 40). This indicates that although the decline in CRF has been restrained globally, it continues to decline in some countries.

We found that there was a small improvement for boys and a negligible change for girls in power. The results of other domestic studies were more negative compared with our study (2, 33), which may be due to different research methods and the improvement of physical fitness for children and adolescents nationwide in recent years. A review on standing long jump performance of 10,940,801 children and adolescents from 29 countries showed negligible international improvement from 1960 to 2017 (16) and a 38% improvement in international sit-up performance from 1964 to 2017 (15), similar to our findings. Changes in flexibility are not consistent between boys and girls. Decrease in flexibility in boys and increase in girls was found in Hong Kong (8) and Slovenia (38), decreases in Poland for both sexes (41), and slight increases in Germany for both sexes (42). The trend of flexibility is not yet consistent across countries, but it can be found that girls perform relatively better.

A systematic review by Fühner et al. (42) found an increase in speed for adolescents worldwide since 2002. Other unquantified systematic reviews have shown inconsistent speed trends across countries (43, 44). In addition, we found significant differences between countries in items tested for speed. Short-distance sprints (e.g., 30/50/60-m runs) are associated with the ability to move quickly, and speed agility (e.g., 10 × 5-m shuttle runs) also includes the ability to react quickly to stimuli and to change body position/direction (11). A study in Italy chose three different programs to test speed, and they found that performance in short-distance sprints stabilized over the last 30 years, but performance in shuttle runs declined (45). Therefore, we estimate that differences in temporal changes in speed across countries may be related to different programs, although speed is highly genetically determined (43). In short, current trends in worldwide speed vary across countries.

Explanations about trends in physical fitness may be related to several factors, including social, behavioral, physical, psychosocial, and physiological factors (15, 16). This paper found that there a large increase in weight and BMI in both sexes. Previous studies have shown an inverted U-shaped relationship between BMI and physical fitness, suggesting that malnutrition and overweight/obesity may have a negative impact on physical fitness (2, 18). Roth (46) reported that taller people should also be allowed a higher BMI to be considered fit. This paper revealed a concurrent increase in height and BMI, which suggested that a portion of the increase in BMI might be beneficial. Unfortunately, the threshold for this increase in this paper was currently unknown and needed to be explored in additional research. Moreover, this study was unable to provide the trend of the prevalence of overweight/obesity, and some studies using the same survey may provide some evidence. Since the reform and opening up in 1978, China’s economy, diet and social structure have been transformed, and people’s living conditions have improved significantly. According to a survey from CNSSCH, the prevalence of thinness among Chinese children and adolescents decreased from 16.4% in 1985 to 2.3% in 2014 (47) and continued to decrease through 2019 (48). The prevalence of overweight and obesity increased from 1.2% in 1985 to 23.4% in 2019, an increase of 18.1 times, and the prevalence of obesity increased from 0.1% in 1985 to 9.6% in 1985, an increase of 75.6 times (48). This could be the reason for the decline in physical fitness. Meanwhile, CRF declined significantly, which may be due to the stronger correlation between body size/composition and CRF. Previous studies have estimated that the increased prevalence of overweight and obesity in the last few years explains 35% ~ 70% of CRF performance in children and adolescents (37).

Trends in physical fitness performance may be influenced by concurrent trends in biological maturation (16, 37), which, along with trends in body size, are thought to be influenced by improved standards of living (e.g., nutrition, education, and income) and more effective prevention and treatment of disease, although it is difficult to quantify the impact of these factors on trends in physical fitness. Older children and adolescents typically perform better than younger counterparts (49), which may be due to increased physical and neuromuscular maturity. A CNSSCH survey showed that the age at spermarche for Chinese boys and the age at menarche for Chinese girls declined from 1985 to 2019 (50, 51), which may be related to the decline in physical fitness in adolescents. The relationship between trends in maturation and trends in physical fitness performance may be mediated by psychosocial factors (52). Girls may be more socially self-conscious during adolescence and therefore more susceptible to peer responses to maturing bodies, decreasing physical activity participation and negatively affecting physical fitness (52, 53). The rate of inadequate moderate to high intensity physical activity (MVPA) among Chinese adolescents aged 13–18 years was 83.4% in 2010 and increased to 83.8% in 2019 (54). MVPA was positively associated with CRF, whereas sedentary time (ST) was negatively associated with CRF (55), and the decline in physical activity level may also be one of the reasons for the decline in physical fitness, especially for CRF.

We also found relatively positive trends in physical fitness among adolescents aged 13–15 years compared to those of adolescents aged 16–18 years. Recognizing the specificity of schools in promoting adolescent health, the Chinese government used “examinations” as a mandatory tool to promote physical fitness among Chinese adolescents. The most typical of these is the Physical Education Entrance Examination for Senior High School (PEESHS) (56). Piloting from the 21st century, most districts in China started the PEESHS in 2007 (57), signifying the full implementation of the policy. Surveys from CNSSCH show that the proportion of Chinese adolescents aged 13–15 and 16–18 with excellent and good physical fitness status has declined by 2.7, 18.5 and 47.1%, 46.7%, respectively, over the past 30 years, and the attainment rate of adolescents aged 13–15 has been higher than that of adolescents aged 16–18 (58, 59). As Wang (60) found that in the past 30 years, Chinese adolescents’ academic burden has become increasingly heavier, physical education classes have been marginalized, and technological advances (Internet, smartphones, video games, etc.) have compressed the time for adolescents to engage in physical exercise, and the lack of accessibility to sports places has limited their participation in physical exercise. These factors have a tendency to have an increased role with age, as demonstrated by the fact that adolescents’ physical exercise behavior decreases with age (61).

This study is the first to investigate temporal changes in the physical fitness of Chinese adolescents over the past three decades. We collected data from repeated cross-sectional national surveys that were representative of the population and used a trained measurement team. We include potential demographic characteristics in our estimates, while stratified analyses helped to control for confounding effects of sex and age. We also estimated changes in the mean body size/composition of children and adolescents, providing some support for the interpretation of trends in physical fitness. This study has several limitations. First, because we could only estimate temporal changes from descriptive data, we were unable to statistically adjust for trends in underlying mechanistic factors (e.g., physical activity level, biological maturity). In addition, our study focuses on long-term trends in physical fitness among in-school adolescents and does not include analyses of the out-of-school group, which prevents us from extending the results to the entire population of Chinese adolescents.

## 5 Conclusion

The height, weight and BMI of Chinese adolescents aged 13–18 years increased significantly from 1985 to 2019. Except for a significant decrease in CRF, the other components of fitness changed little or improved slightly, which may lead to a decline in the health of the population in the future. These trends may have been influenced by nutritional status, biological maturity, physical activity levels or national policies. Recent national efforts to promote physical fitness levels in adolescents have begun to bear fruit, and there is still a need to continue to implement and pursue health promotion strategies to improve the health of the population. We also recommend that physical fitness improvement measures be incorporated into national health surveillance systems to help monitor the progress of physical fitness and public health policies that have been implemented.

## Data availability statement

Publicly available datasets were analyzed in this study. The data analyzed during our study from CNSSCH have been published openly and are freely available. The references where these data can be found are: references 19–26.

## Ethics statement

This study was based on publicly available datasets. Ethical review and approval was not required for the study, in accordance with the local legislation and institutional requirements.

## Author contributions

HR: Data curation, Formal analysis, Methodology, Visualization, Writing – original draft, Writing – review & editing. RT: Funding acquisition, Project administration, Resources, Supervision, Validation, Conceptualization, Software, Writing – review & editing.
